# Cohort Profile Update: 2004 Pelotas (Brazil) Birth Cohort Study. Body composition, mental health and genetic assessment at the 6 years follow-up

**DOI:** 10.1093/ije/dyu144

**Published:** 2014-07-25

**Authors:** Iná S Santos, Aluísio JD Barros, Alicia Matijasevich, Roberta Zanini, Maria Aurora Chrestani Cesar, Fabio Alberto Camargo-Figuera, Isabel O Oliveira, Fernando C Barros, Cesar G Victora

**Affiliations:** ^1^Universidade Federal de Pelotas, Pelotas, Brazil, ^2^Universidade de São Paulo, São Paulo, Brazil and ^3^Universidade Católica de Pelotas, Pelotas, Brazil

## Abstract

This is an update of the 2004 Pelotas Birth Cohort profile, originally published in 2011. In view of the high prevalence of overweight and mental health problems among Brazilian children, together with the availability of state-of-the-art equipment to assess body composition and diagnostic tests for mental health in childhood, the main outcomes measured in the fifth follow-up (mean age 6.8 years) included child body composition, mental health and cognitive ability. A total of 3722 (90.2%) of the original mothers/carers were interviewed and their children examined in a clinic where they underwent whole-body dual X-ray absorptiometry (DXA), air displacement plethysmography and a 3D photonic scan. Saliva samples for DNA were obtained. Clinical psychologists applied the Development and Well-Being Assessment questionnaire and the Wechsler Intelligence Scale for Children to all children. Results are being compared with those of the two earlier cohorts to assess the health effects of economic growth and full implementation of public policies aimed at reducing social inequalities in the past 30 years. For further information visit the programme website at [http://www.epidemio-ufpel.org.br/site/content/coorte_2004/questionarios.php]. Applications to use the data should be made by contacting 2004 cohort researchers and filling in the application form available at [http://www.epidemio-ufpel.org.br/site/content/estudos/formularios.php].

**Key Messages**

- The study showed that it is possible to recruit a population-based cohort and to follow up over 90% of all participants prospectively for 6 years.
- Some of the most interesting results arise from the comparisons with the 1982 and 1993 cohorts carried out in the same population. The most impressive findings included the marked increase in preterm deliveries (from 6.3% in 1982 to 14.7% in 2004), reductions in child undernutrition and a marked increase in child overweight over time.
- Social inequalities in access to services and to child health indicators still persist, in spite of the evidence of improvement over time.

## What is the rationale for the new data collection?

This multi-ethnic population-based study is being conducted in the city of Pelotas in Southern Brazil (http://www.ncbi.nlm.nih.gov/pubmed/20702597).^1^ All births occurring in Pelotas, from 1 January to 31 December 2004, were enrolled and followed up.^2^ Mothers were interviewed soon after delivery (perinatal study) using a standardized questionnaire, which was planned to be compatible with those used in two previous population-based cohorts (1982 and 1993) in the same city and to expand upon these by also addressing emerging health and developmental problems.^1^ The main objectives of the original study were to investigate the impact of early life exposures (such as: antenatal and perinatal conditions; maternal socioeconomic, demographic and environmental characteristics; breastfeeding; development; infections; accidents; and healthcare access, use and financing) on health outcomes and to study inequities in health conditions.^2^ Since the inception of the cohort, Brazil has entered a period of intense economic growth. In addition, the government has prioritized policies for reducing poverty and social inequalities, mainly through a countrywide conditional cash transfer programme (Programa Bolsa Família). Meanwhile, prevalences of obesity and mental health problems are increasing among Brazilian children.^3^^–^^7^ Some researchers fear that a sudden increase in family income may favour the consumption of high-calorie foods such as sugary drinks and snacks. Life course research can help assess the effects of such changes on the lives of children. This paper describes the methods and some of the findings from this fifth visit to the cohort, which was planned mainly to assess body composition, mental health, development and cognitive ability. The reason for changing the type of data collected, moving from anthropometry alone to anthropometry together with state-of-the-art equipment for measuring body composition, was to understand the role of early life determinants in body fat mass accumulation in childhood, in terms of both quantity and distribution.^8^ Furthermore, the use of diagnostic instead of screening tests for evaluation child mental health was an improvement in comparison with previous follow-ups.

## What will be the new areas of research?

The main outcomes in the fifth follow-up wave included child nutritional status and body composition, mental health, development and cognitive ability. Taking advantage of the data collected in previous follow-up visits, we plan to analyse the separate and combined effects of genetic factors, socioeconomic trajectories and early life exposure to important determinants such as preterm birth, intra-uterine growth restriction and infant feeding practices. Specifically, the aim of the fifth follow-up was to investigate the association between those earlylife determinants and body composition and mental health at the age of 6 years.

## Who is in the cohort?

**Figure 1.**
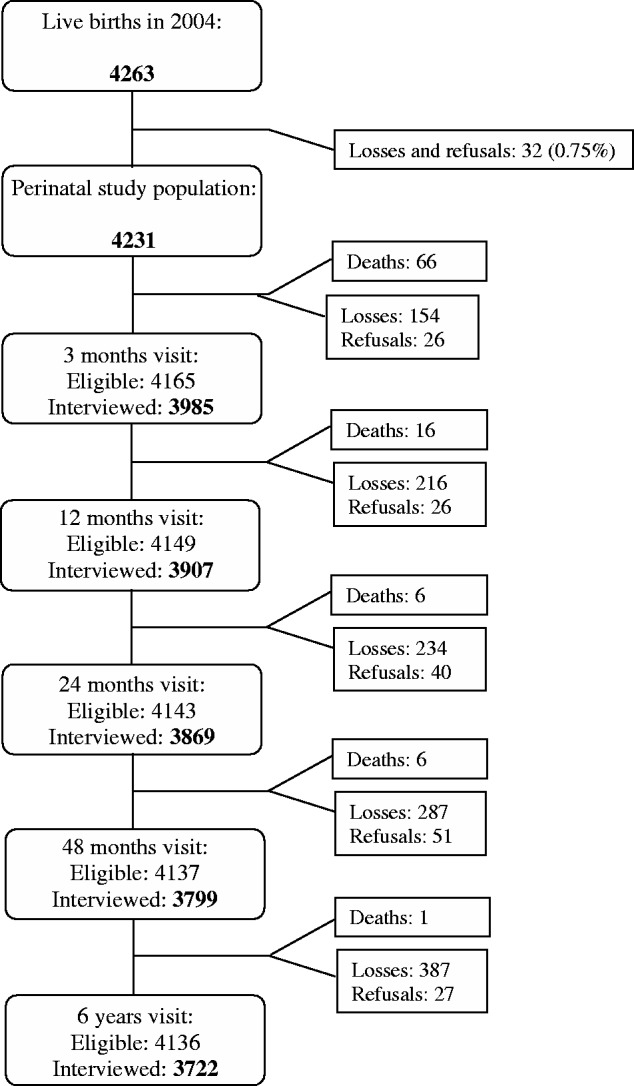
Flow chart of the 2004 Pelotas Birth Cohort.

The cohort flow chart in Figure 1 shows the number of children enrolled in the cohort and the number followed up at each visit. A total of 95 children had died and 3722 of the 4231 live-birth children were visited during the fifth follow-up wave. The proportions of children traced at the 6 years follow-up according to maternal socioeconomic and demographic characteristics and by child sex and birthweight are shown in Table 1. Follow-up rates were at least 87% in all subgroups, except for the small group of children born to mothers without any education of whom 83.7% were located. Follow-up rates were lower among those from families in the upper (88.8%) and lower extremes of the income distribution (87.2%). There were no differences in follow-up rates according to maternal self-reported skin colour.

**Table 1.** Socio-demographic characteristics of mothers and children enrolled in the 2004 Pelotas Birth Cohort, and percentage located at the last follow-up (6 years)

Characteristics (%)	Original cohort (N=4231)	Percentage located	*P*
Maternal age (years)			0.025
<20	799 (18.9)	91.4	
20–34	2865 (67.8)	89.4	
≥35	563 (13.3)	92.7	
Maternal education (years)			0.059
0	43 (1.0)	83.7	
1–4	612 (14.6)	88.2	
5–8	1731 (41.4)	91.3	
≥9	1802 (43.0)	89.8	
Self-reported maternal skin colour			0.43
White	3090 (73.0)	90.4	
Black or brown	1141 (27.0)	89.6	
Family income (quintiles)			0.0013
1 (poorest)	872 (20.6)	87.2	
2	855 (20.2)	91.1	
3	816 (19.3)	91.4	
4	858 (20.3)	92.4	
5 (wealthiest)	830 (19.6)	88.8	
Birth weight (g)			0.34
<2500	424 (10.0)	89.4	
2500–3499	2694 (63.7)	89.8	
≥3500	1110 (26.3)	91.3	
Sex			0.26
Boys	2196 (51.9)	90.7	
Girls	2035 (48.1)	89.6	
Preterm birth			0.86
Yes	612 (14.5)	90.0	
No	3603 (85.5)	90.3	

## What has been measured?

Detailed description of variables collected from birth to 4 years follow-up can be found in a previous publication^1^ Differently from previous follow-ups of the three cohorts, interviews at 6 years were conducted with the help of personal digital assistants instead of printed questionnaires, and children were examined at a clinic where all the equipment was installed. Detailed assessments of health, diet and lifestyle of the families, housing conditions, health behaviours, medical care and quality of life of children and their mothers were gathered. Anthropometric measurements, assessment of body composition and physical activity, spirometry and blood pressure measurement were performed. History of accidents and injuries was explored, and tests for cognition and mental health evaluation were applied for all children.

For dietary evaluation, the mothers answered a food frequency questionnaire covering the child’s annual consumption of 54 food items. The World Health Organization Quality of Life Questionnaire (WHOQOL-BREEF)^9^ and the Edinburgh Postnatal Depression Scale (EPDS) (in a Portuguese version validated in Brazil)^10^ were answered by mothers. To ascertain the presence and degree of child maltreatment, the Parent-Child Conflict Tactics Scales (CTSPC)^11^ was applied for the mothers.

Children’s mental health was assessed by clinical psychologists through maternal report using the online Development and Well-Being Assessment (DAWBA) questionnaire validated in Brazil by Fleitlich-Bilyk & Goodman.^12^ Presence of psychiatric disorders was classified according to DSM-IV and ICD-10 classifications.^13^^,^^14^ Four subtests of the Wechsler Intelligence Scale for Children (WISC III)^15^ (picture completion, block design, arithmetic and picture concepts) were applied for the children by trained psychologists. Estimates for intelligence quotient (IQ) using both the American and the Argentinian scoring were generated.

Child anthropometric measurements included body weight, standing and sitting height, subscapular and tricipital skinfolds and abdominal and arm circumferences. Maternal and child weight, as well as percentages of body, trunk and members fat in children, were measured with a digital scale (Tanita® BC-558 Ironman Segmental Body Composition Monitor, maximum 150 kg and 100 g precision). Child abdominal and arm circumferences were taken with a non-extensible tape (Cardiomed® Mabbis). Maternal and child heights were taken with a stadiometer (Harpenden®) (maximum 2.06 m and 1 mm precision). Mothers and children who were visited at home had their height measured with a portable stadiometer (Alturexata®, maximum 2.13 m and 1 mm precision). If these participants came to the clinic on a later occasion, measures were repeated using the clinic equipment.

Body composition and shape were assessed by air-displacement plethysmography^16^ (Bod Pod®), whole-body dual-energy X-ray absorptiometry^17^ (Lunar Prodigy, GE Healthcare®) and 3-dimensional photonic scan^18^ (3D Photonic Scanner TC^2^®). Blood pressure was measured with the digital automatic OMRON sphygmomanometer (model HEM 742) using the right arm with the child seated after at least 5 min rest. Appropriate cuffs for children (arm circumferences ≤23 cm or >23 cm) were used. Spirometry was performed with a portable spirometer (trade mark NDD, EasyOne®). Samples of saliva were collected for DNA extraction and future analyses. The DNA Oragene Genotek® – 250 kit was used for sample collection and storage. Accelerometer devices for physical activity measurement were worn by 3331 children 4-6-days a week.^19^

## What has it found? Key findings and publications

A summary of the results published up to 2010 is available in the original cohort profile^1^ and a complete list of articles may be found at [http://www.epidemio-ufpel.org.br/blog/estudos-de-coortes/coorte-2004]. Analyses conducted since the publication of the original profile addressed inequalities in health, breastfeeding, mode of delivery and maternal behaviours, among others.^19–41^ Results of analyses on body composition, conditional growth, mental health and IQ with data from the 6 years follow-up are briefly described below as examples of potential analyses of our large dataset.

### Child body composition

Body composition assessed by air-displacement plethysmography at the age of 6 years showed that girls had higher mean adiposity measures and lower mean fat-free mass (FFM) than boys (Table 2). Fat mass (FM) in girls showed on average an excess 0.7 kg of fat compared wwith boys. After dividing by height squared (fat mass index or FMI), this difference was smaller, with a mean value of 0.6 kg/m^2^ greater in girls. For FFM and fat free mass index (FFMI), boys had higher means (1.0 kg and 0.4 kg/m^2^ higher, respectively,) than girls.

**Table 2.** Means and standard deviations of anthropometric variables and body composition measures provided by air-displacement plethysmography (BodPod) at 6 years of age. The 2004 Pelotas Birth Cohort. (N=3350)

	Girls		Boys		
Variable (unit)	N	Mean ± SD	N	Mean ± SD	*P*
Weight (kg)	1619	24.8 ± 6.1	1731	25.1 ± 5.8	0.1206^a^
Height (m)	1602	1.20 ± 0.06	1709	1.22 ± 0.06	<0.0001^a^
FM^c^ (kg)	1619	6.6 ± 3.8	1731	5.9 ± 3.6	0.0001^a^
FMI^c^ (kg/m^2^)	1602	4.5 ± 2.3	1709	3.9 ± 2.1	0.0001^a^
% FM^c^ (%)	1619	25.1 ± 7.9	1731	22.2 ± 7.9	<0.0001^b^
FFM^c^ (kg)	1619	18.2 ± 2.8	1731	19.2 ± 2.9	0.0001^a^
FFMI^c^ (kg/m^2^)	1602	12.5 ± 1.1	1709	12.9 ± 1.2	< 0.0001^b^
% FFM^c^ (%)	1619	74.9 ± 7.9	1731	77.8 ± 7.9	< 0.0001^b^

FMI, fat mass index; % FM, percentage of fat mass; FFM, fat-free mass; FFMI, fat-free mass index; % FFM, percentage of fat-free mass.

^a^Test of homogeneity (non-parametric).

^b^Test of homogeneity (parametric).

^c^Fat mass.

Additional analyses showed that mean FM, %FM and FMI in both girls and boys increased with socioeconomic status (SES) and maternal education (data not shown). Mean FFM increased in the same direction, whereas %FFM showed an inverse association with greater values among poor children and those born to less educated mothers. Skin colour was classified as white, brown or black, following the categorization used by the Brazilian census institute (IBGE). Mean adiposity measures were higher among white-skinned children in comparison with black- or brown-skinned children. The %FFM was greater among black- or brown-skinned children.

### Conditional growth and body composition at 6 years of age

Analyses are under way, using conditional growth methods to attempt to disentangle the roles of linear growth and weight gain, among different age ranges from birth to 4 years of age, in body composition at the age of 6 years. Compared with the existing literature,^42^ our analyses have the advantage of using as outcomes measures of body composition obtained through DXA and BodPod, as opposed to less precise measures based on skinfolds or bioelectrical impedance analysis (BIA).

### Mental health

The analyses highlighted the early onset of psychiatric disorders: prevalence of at least one diagnosis of psychiatric disorder according to DSM-IV and ICD-10 classifications was 13.2% [95% confidence interval (CI) 12.2; 14.4%] and 12.8% (11.7; 13.9%), respectively.^43^ Anxiety disorders were the most prevalent (9%). Psychiatric disorders were more common among boys than girls (14.7% vs 11.7%). Both sexes showed very similar prevalence of depressive and anxiety disorders. Attention deficit / hyperactivity disorder (ADHD) was more prevalent among boys than girls (3.4% vs 1.8%) as well as oppositional defiant/conduct disorders combined (3.7% vs 1.5%).

Children from low-income families had higher prevalence of any mental disorder than those from wealthy families (14% vs 8%), with externalizing disorders (attention deficit / hyperactivity disorder and oppositional defiant/conduct disorder) being more frequent among the poorest children. Depression and anxiety disorders had similar frequencies across SES categories.

### Intelligence quotient

Prevalence of low IQ (score below -1 standard deviation from the cohort mean value at the age of 6 years) was 16.9% (95% CI 15.6; 18.1%). Multivariable analyses identified socio-demographic characteristics (male sex, black or brown skin colour, low maternal schooling, mothers who did not work outside the home in the first 12 months post-partum, low SES and 3+ persons/bedroom), parental smoking, weight or height for age deficits, short breastfeeding duration and negative maternal assessment of child health as predictive factors of low IQ at 6 years of age. Children with such characteristics can be easily identified by healthcare providers and receive interventions aimed at stimulating psychomotor development.

## What are the main strengths and weaknesses?

One of the main strengths of the 2004 cohort is the potential for comparative analyses across the other two Pelotas birth cohorts, a unique opportunity to assess changes in health exposures and outcomes over time in the same city. The assessment of body composition among 6-year-olds using traditional (anthropometry) and state-of-the-art methods (DXA, BodPod and 3-D photonic scan) is essential for current and future analyses of the determinants and consequences of child overweight, and tracking from childhood to adult life. Extraction of DNA from saliva samples and their storage in a biobank will allow future genome-wide (GWAS) and full genome analyses, as well as epigenetic studies.

Detailed information on child development and cognitive ability data including IQ, and mental health including application of a thorough diagnostic test (DAWBA) to almost all (3585) of the 3722 participants, together with screening for maternal symptoms of anxiety and depression, provide a huge amount of information to explore determinants, natural history and prognostic factors for current and future mental health.

**Figure 2.**
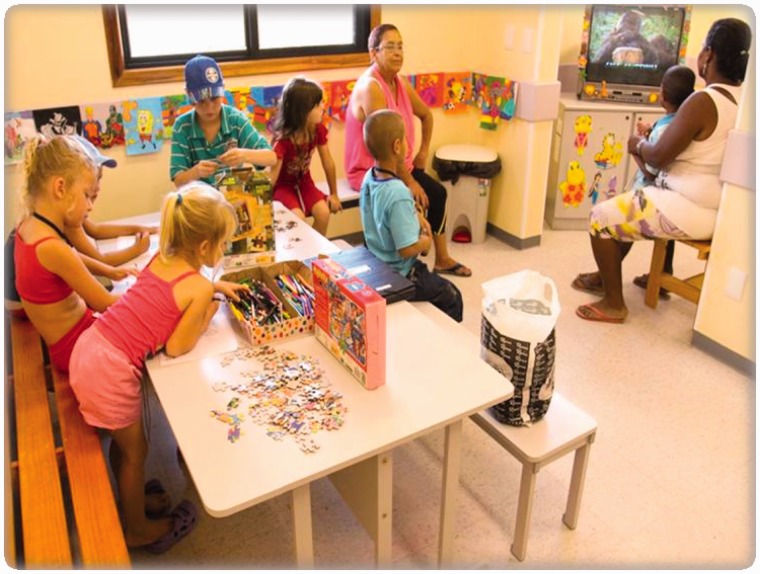
Children of the 2004 Cohort at the recreation room during the fifth follow-up (2010-11).

The principal limitation of the 6 years follow-up was the time required for the simultaneous assessments of mothers and children during a single visit to the clinic. During the data collection phase, several logistical adaptations had to be made in order to avoid queues and reduce the time mothers and children spent in the clinic. Even so, visits lasted on average 3 h. We attempted to make the visit a pleasant experience, by offering healthy snacks, and leisure activities were offered to children under the supervision of our the clinic staff, in a specific recreation room (Figure 2).

## Can I get hold of the data? Where can I find more?

Joint analyses of the cohort data are welcome and we have collaborated successfully with investigators from the UK (University of Bristol) and with Chile and Mexico through a Latin American cohorts consortium. Exchange of doctoral or postdoctoral fellows between other institutions and Pelotas is very welcome. For young researchers from low- and middle-income countries, there are sponsored postgraduate positions available on a competitive basis. Since 2005, a Wellcome Trust-supported programme has trained 25 MSc and PhD students from Latin America and Africa. For further information on postgraduate training, check the programme website at [http://www. epi demio-ufpel.org.br/projetos_de_pesquisas/estudos/coorte_ 2004] or e-mail the investigators involved in the research areas of interest. The questionnaires and interviewer guides from all follow-up visits are available in electronic formats at [http://www.epidemio-ufpel.org.br/site/content/coorte_2004/questionarios.php]. Applications to use the data should be made by contacting the researchers of the 2004 cohort and filling the application form for the Pelotas Birth Cohorts available at [http://www.epidemio-ufpel.org.br/site/content/estudos/formularios.php].

## Funding

The 2004 birth cohort study is currently supported by the Wellcome Trust through the programme entitled Major Awards for Latin America on Health Consequences of Population Change (Grant no. 086974/Z/08/Z). The World Health Organization (Grant no. 03014HNI), National Support Program for Centers of Excellence (PRONEX) (Grant no. 04/0882.7), Brazilian National Research Council (CNPq) (Grant nos. 481012-2009-5; 484077-2010-4; 470965-2010-0; and 481141- 2007-3), Brazilian Ministry of Health (Grant no. 25000.105293/2004-83), and Children's Pastorate have supported previous phases of the study.
